# Psychological well-being and employment status during the COVID-19 pandemic

**DOI:** 10.1192/j.eurpsy.2021.685

**Published:** 2021-08-13

**Authors:** P. Valenzuela, C. Barrientos, F. Molina, D. Valdés, I. Leniz, G. Reginatto, A. Basaigoitia, M. Solis-Soto, M. Burrone

**Affiliations:** 1 Instituto De Ciencias De La Salud, Universidad de O’Higgins, Rancagua, Chile; 2 Dirección De Asuntos Estudiantiles, Universidad de O´Higgins, Rancagua, Chile; 3 Consulting Office, Salud Global, Sucre, Bolivia

**Keywords:** psychosocial, COVID-19, Chile, mental health

## Abstract

**Introduction:**

Several restrictive measures have been implemented to reduced COVID- 19 impact with unknown consequences on people daily life.

**Objectives:**

The primary objective is to asses the psychosocial impact and employment status changes since lockdown COVID-19 measures in Chile.

**Methods:**

Cross-sectional study was implemented using an anonymous and self-administered online questionnaire. Adult people were invited to participate through social networks between May to June 2020. The questionnaire included sociodemographic information, coping strategies, changes in income and working conditions and psychological distress (K10 Scale).

**Results:**

3102 participants over 18 years answered the questionnaire. 69.9% reported psychological distress mainly women (82.2%), members of the public health system (59%), dependent workers (39.8%), people who suffered income reduction (36.8%)., and those who changed their employment status (26.4%). Participants who presented income reduction were 1.83 times more likely to present psychological distress than those without changes (p <0.001)

**Conclusions:**

Pandemic crisis had impacted population health, especially in some specifics groups that could be targeted for future interventions.

